# Facts Tending to Show That the Anterior Columns of the Spinal Cord Serve to Transmit Impressions of Sensation

**Published:** 1859-06

**Authors:** 

**Affiliations:** Aggrégé Honoraire à la Faculté de Médecine de Paris, Médecin Des Hôpitaux, etc.


ART. V.—*Facts tending to show that the Anterior Columns of the Spinal Cord serve to Transmit the Impressions of Sensation.* By DR. NONAT. *Aggrégé Honoraire à la Faculté de Médecine de Paris, Médecin Des Hôpitaux, etc.* Letter to Dr. E. Brown Séquard.* Translated by LUCIEN DAMAINVILLE, Medical Student, Buffalo, N. Y.

PARIS, *June* 18, 1856.

SIR AND HONORED *Confrère* :—

As is announced by the "*Gazette Médicale*" of 1837, I have read before the Academy of Medicine a paper in which I have endeavored to demonstrate that the anterior columns of the spinal marrow serve to transmit sensitive impressions.

This paper is entitled : "*To what extent can we admit special nerves for motion and for sensation?*" Examining this question, I was naturally led to investigate whether the columns of the spinal marrow

* It is more than two years since I received this letter from Dr. Nonat, as an answer to a few questions which I had addressed to him about his experiments mentioned in one or two lines in the *Gazette Médicale*, of 1837. I had proposed to myself to publish this answer in a work upon the Physiology and Pathology of the nervous system, which work I could not publish till the present date, on account of journeys which I was compelled to make, and other pressing circumstances. Although there is nothing entirely new in the experiments of the eminent physician, the author of this letter, and although perhaps one of the opinions enunciated in it does not agree exactly with the facts, I think it my duty to publish this,—in the first place, to do justice to the author; in the second place, to add to the testimony of Bellingeri, Schœps, Mr. Calmeil, Rolando, Seubert, Van Deen, Budge, Stilling, possess the same speciality of function as the roots of the nerves which grow out of them.

I have made, upon this subject, a series of experiments upon living animals, and was able to convince myself that the anterior and posterior columns of the cord may serve to transmit, at the same time, sensation and motion, and that consequently their functions are not so special as those of the roots of the spinal nerves.

I had proposed to myself to publish my work, and I have not done it till now, to my great regret.

Allow me to communicate to you the passage pertaining to the subject in question.

After having confirmed by numerous experiments the opinion of Magendie about the sensibility of the anterior and posterior columns; after having re-demonstrated that a stylet may be introduced in the spinal marrow without the animal giving any sign of sensibility,—I expressed myself in the following manner :—

Because the anterior columns of the cord are almost insensible, does it ensue that they are unable to transmit sensations? I do not believe it ; for the peduncles of the cerebrum are themselves deprived of sensibility, and still they propagate the sensations of the medulla oblongata to the cerebrum.

In order to demonstrate that the anterior columns of the spinal cord do not play any part in the transmission of sensation, it was to be tried if the destruction of the posterior columns of the cord is followed by abolition of sensation in the parts corresponding to them. Although it is difficult to make a section of the posterior half of the cord without wounding the anterior columns, still I was able to convince myself that motion and sensation persisted, even when the posterior and a part of the anterior columns were incised. The same thing exists in regard to the section of the anterior columns of the cord ; it is not followed by the complete loss of motion in the parts receiving their nerves from below the incision.

---

and many experimenters of less reputation than the preceding ones, the competent testimony of a man of rare good faith, and still rarer modesty. We will refer the reader, who wishes to know in what way our opinions agree and disagree with those of our learned colleague, to the volume of the Memoirs of the Biological Society for 1855, to two articles inserted in the first number of this journal (pp. 139 and 176), to a translation which is to be found in the present number, and finally to our lectures which have been published since last July 3d, in the London Lancet, and which give, with a great many details, the lessons that we had the honor to give at the Royal College of Surgeons of London, and in presence of a great number of surgeons and physicians of distinction.

These results, which I have verified several times, prove to us that although the anterior columns are designed to transmit motion, they may also serve to conduct external impressions. It is, indeed, in accordance with certain pathological facts, in which motion and sensation have been seen to persist in the lower extremities, notwithstanding the deep alteration of the anterior columns of the marrow, above the lumbar enlargement. For instance, in man, as well as in animals, as long as the spinal marrow is not disorganized in its whole thickness, motion and sensation may still survive in the parts receiving their nerves below the lesion of the marrow. But the movement is constantly abolished in the parts corresponding exactly to the softening of the anterior columns of the spinal cord. Suppose this lesion to exist on a level with the brachial plexus, the motion will be destroyed in the thoracic extremities.

In a word, every time one part of the body communicates no more with the nervous centres, except by the posterior roots of the spinal nerves and posterior columns of the cord, it is deprived of the faculty of motion. From this we are authorized to conclude, that nature did not separate the motor and sensitive functions of the columns of the cord in such a complete manner as those of the roots of the spinal nerves.

Such is, sir and honored *confrère*, the account of the experiments that I have made in regard to the speciality of the functions devolved upon the anterior and posterior columns of the cord.

You see that my results are in harmony with those that you obtained subsequently.

I am grateful to you for having thought of me, and pray accept, &c.. &c.

